# A Virtual Success, Online Conferences Provide Similar Educational Benefit to In-Person Events

**DOI:** 10.1177/15533506211056149

**Published:** 2022-03-27

**Authors:** Conor Hennessy, Devon Brameier, Matthew Williams, James McVeigh, Marta de Andres Crespo, Solveig Hoppe, Ashok Handa

**Affiliations:** 1 Nuffield Department of Surgical Sciences, 6396 University of Oxford, Oxford, United Kingdom

## To the Editor:

Following the release of the ‘Future of Surgery Report’ by the Royal College of Surgeons of England (RCSE), we set out to provide a platform for the dissemination of knowledge surrounding surgical innovation. The RCSE report discussed the important role of technology in the future of surgery, and stated that exposure to technological developments and surgical innovation should take place early on in medical education to ensure that future surgeons have the broadest skillset possible.^1^

This report inspired the establishment of the Oxford Surgical Innovation (OxSI) Conference, an annual conference focused on the promotion of safe and successful innovation in surgery. We have previously described the success of the conference in its inaugural event in 2019,^2^ as well as the second annual event in 2020.^3^ We sought to build on the success of the previous 2 years and deliver a third conference highlighting cutting-edge surgical innovation through a series of lectures and workshops.

The 2021 OxSI Conference was originally planned as a physical conference in St Catherine’s College, Oxford, in April of 2021. However, due to the impact of the COVID-19 pandemic, the decision was made to host the conference virtually. The event consisted of 5 keynote speaker presentations and 5 workshops, of which 2 could be selected by delegates. We sought to achieve 2 goals:

1. Educate the attendees to the practicalities of engaging in surgical research and innovation.
2. Showcase examples of ongoing innovation in surgical research.

Feedback from delegates was collected using a four-point Likert scale. The virtual nature of the conference led to concerns that the educational and interactive value of an in-person event may be lost. However, the feedback was overwhelmingly positive, with average scores of 3.58 for the plenary sessions and 3.54 for the workshops. This was in keeping with the scores from last year’s event, which were 3.60 and 3.50, respectively, with no significant difference observed when compared statistically (*P* > .05).^3^ Furthermore, we saw significant improvements in our 2 key metrics of interest following the post-conference questionnaire, with statistically significant increases in understanding the practicalities of engaging in safe surgical innovation (pre = 2.70, post = 3.43, +.73 (*P* < .001)) and in awareness of current innovations in surgical practice (pre = 2.77, post = 3.42, +.65 (*P* < .001)). These data suggest that the virtual format of the conference provided the same educational benefit to the delegates as the previous in-person iterations of OxSI.

One welcomed benefit from the switch to virtual delivery was the increased reach of the conference. OxSI 2021 hosted delegates from multiple countries outside the United Kingdom, including the USA, Brazil, Russia, Kenya, Germany and Ireland. The comparison of the conference outcomes in 2020 and 2021 suggest that an online conference provides equal benefit as an educational tool as in-person events. It also has the added benefits of increased accessibility, allowing us to attract a wider audience. Given the success of the virtual conference this year, as well as the previous successful events, we plan to conduct the future OxSI events using a hybrid model. The combination of a physical conference with virtual elements, such as livestreamed plenaries and workshops, would allow continued international attendance in conjunction with the benefits of in-person networking and interaction. We believe the virtual model of conferences will persist in some form in the aftermath of COVID-19, as there are many benefits to the new online models we have seen develop over the last 18 months.
